# Does occupational exposure to low-dose ionizing radiation affect bone marrow thrombopoiesis?

**DOI:** 10.1186/1755-7682-4-8

**Published:** 2011-02-23

**Authors:** Douaa Sayed, Mostafa E Abd Elwanis, Saly Y Abd Elhameed, Hanan Galal

**Affiliations:** 1 Flow Cytometry Lab., Department of Clinical pathology, South Egypt Cancer Institute, Assiut University, Assiut, Egypt; 2 Department of Radiotherapy, South Egypt Cancer Institute, Assiut University, Assiut, Egypt; 3 Department of Forensic Medicine and Clinical Toxocology, Faculty of Medicine, Assiut University, Assiut, Egypt; 4 Department of Clinical pathology, Faculty of Medicine Assiut University, Assiut, Egypt

## Abstract

**Background:**

The biological effects of high levels of radiation exposure are fairly well known, but the effects of low levels of radiation are more difficult to determine because the deterministic effects do not occur at these levels.

**Methods:**

In order to assess the risk of this exposure on BM thrombopoiesis, we measured reticulated platelets (RP) by flow cytometry in 14 hospital workers (12 technicians and 2 nurses) exposed to low level ionizing radiation in Radiotherapy Department in South Egypt Cancer Institute.

**Results:**

There are significant difference in the percentage of RP in the peripheral circulation of the workers (*p *= .008) and no significant difference in the proportion of other blood elements in the peripheral circulation.

**Conclusions:**

We think that measuring RP by flow cytometry is a rapid, non-invasive method to asses an early affection of thrombopoiesis. This type of monitoring may be used as an indicator to detect early BM affection and to demand more controls in radiation protection.

## Introduction

Evaluation of thrombopoiesis by quantifying reticulated platelets (RP) has been described [1,2]. RP by analogy to reticulocytes, are the youngest circulating platelet population and they contain abundant amounts of mRNA. RP were first described in 1969 [3] by direct visualization from peripheral blood. In 1990, Kienast and Schmitz reported the first measurement of RP by flow cytometry. RP measurement is a non-invasive test that provides indirect information about thrombopoietic activity in bone marrow [4].

A chronic radiation dose is a relatively small amount of radiation received over a long period of time. The body is better equipped to tolerate a chronic dose than an acute dose. The body has time to repair damage because a smaller percentage of the cells need repair at any given time. The body also has time to replace dead or non-functioning cells with new, healthy cells. This is the type of dose received as occupational exposure [5].

The toxicological effects of high levels of radiation exposure are fairly well known, but the effects of low levels of radiation are more difficult to determine because the deterministic effects do not occur at these levels [5].

Since deterministic effects do not generally occur with chronic dose, in order to assess the risk of this exposure, we must look to other types of effects. The risks for these effects are not directly measurable in populations of exposed workers, therefore the risk values at occupational levels are estimates based on risk factors measured at high doses [5].

The purpose of this work was to provide data on the thrombopoiesis affection due to the occupational exposure to ionizing radiation, by noninvasive, sensitive indicator which is RP value assessed by flow cytometry.

## Materials and methods

### Subjects

Blood samples were obtained from 14 hospital workers (12 technicians and 2 nurses) exposed to low level ionizing radiation in Radiotherapy Department in South Egypt Cancer Institute (Table 1). All of them gave written informed consent to participate in this study. Radiation dose accumulated by occupationally exposed over years was calculated on the basis of individual TL-dose records and multiplied with exposure time. All measurements were performed by dosimetry system (HARSHAW TLD 6000 card reader). We have also studied 14 unexposed controls with matched sex and age (7 males and 7 females; age 31 ± 2.7 years). Thorough history and clinical examination and complete blood picture were done for all controls included in this study. Only individuals, without concurrent infections and medications (esp. aspirin) and no general and dental X-rays in the last 6 months were included in the control group.

**Table 1:** Demographic data of 14 hospital workers exposed to low level ionizing radiation

No	Sex	Age	Smoking	Working since	Exposure/day	Instrument	TLD
1	Male	34y	+	3/1998	6hrs	Mould room	0.6
2	Male	32y	-	3/1998	4hrs	Linear	0.86
3	Male	27y	-	4/2002	4hrs	Linear	0.53
4	Male	34y	+	3/1998	4hrs	Linear	0.61
5	Male	32y	+	3/1998	4hrs	Linear	0.99
6	Male	33y	+	3/1998	4hrs	Linear	0.54
7	Male	27y	-	7/2003	4hrs	Linear	0.63
8	Male	33y	+	3/1998	4hrs	Linear	0.32
9	Female	33y	-	1/1998	4hrs	Linear	0.71
10	Female	34y	-	1/1998	6hrs	Simulator	0.98
11	Female	32y	-	1/1998	6hrs	simulator	0.85
12	Female	32y	-	1/1998	6hrs	simulator	0.72
13	Female	33y	-	1/2007	6hrs	Nurse	0.16
14	Female	25y	-	7/2003	6hrs	Nurse	0.43

### Methods

Blood was collected in EDTA tubes. All blood samples were analyzed less than 6 hours after collection. Five μl of whole blood were incubated for 15 min in the dark at room temperature with 5 μl of Per-CP labeled antiglycoprotein III (CD61-PerCP Becton Dickinson SA) and 30 μl of phosphate buffer saline (PBS). A control tube was used for each sample with 5 μl of isotypic mouse control (IgG1-mouse PerCP Becton Dickinson SA). After incubation, 1 ml thiazole orange (TO; Retic-count, Becton Dickinson SA) 1/10 solution in Flow sheath was added to the test tube and 1 ml Flow sheath (Becton Dickinson SA) solution was added to the control tube. After incubation for 1 hour in the dark at room temperature, analysis by flow cytometry was performed immediately using FACSCaliber (BD, USA).

Identifying of platelets according to their characteristic were determined using (log forward scatter) for size and (log side scatter) for granularity. Platelet gate was adjusted such that >95% of the particles analyzed were anti CD61 positive. A dot plot cytogram (CD61-PerCP versus TO fluorescence) was generated, and RP rate was expressed as a percentage of both a TO and CD61-PerCP positive population among 10,000 identified platelets. The threshold of TO fluorescence was chosen so that more than 99% of the CD61-PerCP positive population was negative for TO. In each session, a sample with a normal number of platelets was used as a control [6].

### Statistical analysis

The data were collected, categorized and processed by using Statistical Package for Social Sciences (SPSS), version 15 software package. The quantitative variables were expressed as mean ± standard deviation (SD) and comparison was done using paired students *t*-test. *P*-value levels of <0.05 was considered statistically significant. Correlations between quantitative variables were done using Pearson correlation and multiple regression analysis by stepwise method.

## Results

The laboratory data of all workers and controls are listed in table 2. The present study revealed significant difference in the percentage of RP in the peripheral circulation of the workers exposed to low level ionizing radiation when compared to those of the controls (*p *= .008). In addition, there was no significant difference in the proportion of other blood elements in the peripheral circulation of the workers compared to those of the controls.

**Table 2:** Laboratory data of 14 hospital workers exposed to low level ionizing radiation and 14 controls

	Hb (g/dL)	Hct (%)	MCV (fL)	Platelets (×10^9^/L)	MPV (fL)	RP (%)
Workers	12.8 ± 1.7	38 ± 4.7	81.5 ± 8.2	246.6 ± 44.2	8.3 ± 0.88	12.5 ± 5.5
Controls	12.3 ± 1.8	37.6 ± 4.9	81.8 ± 5.4	239.2 ± 57	8.4 ± 1.3	5.6 ± 2.6
*P *value	0.5	0.9	0.9	0.7	0.8	0.008

Quantitative variables are expressed as mean ± standard deviation

Hb = hemoglobin; Hct = hematocriet; MCV = mean corpuscular volume; MPV = mean platelet volume; RP = reticulated platelets

The percentages of RP in the peripheral circulation of the workers not significantly correlated with other studied variables. All studied variables showed no significant correlation with the TL dose or exposure time.

## Discussion

Exposure to low doses of ionizing radiation is a fact of life in certain occupational settings. Radiation accidents, while unfortunate at the minimum and devastating in the worst cases, will no doubt continue to occur. Fortunately most radiation exposures involve low doses (<1 Gy) and as such do not have immediate life threatening effects. However, long-term effects of low-dose exposures may be real and should be given serious consideration [7].

Since small doses of radiation are known to diminish the effects of subsequent larger doses, a phenomenon that is called adaptive response [8], it is possible that chronic exposure may lead to a state in which some cells have certain cytoprotective genes that are perpetually expressed at elevated levels. The results of such an effect, if true, would mean that the actual dose is underestimated, perhaps by a significant amount [7].

Therefore, it is important to provide data on the actual effect of occupational exposure to ionizing radiation. As the hemopoietic system is the most sensitive biological indicator of radiation exposure [5], we assessed the thrombopoiesis affection due to the occupational exposure to ionizing radiation by measuring RP. We found an increased percentage of RP in the workers than healthy controls. The number of reticulated platelets reflects the rate of thrombopoiesis; increasing when platelet production rises and decreasing when production falls [9,10].

This is in agreement with Fliedner and Graessle [11] who reviewed the role of cell renewal systems in maintaining the integrity of the mammalian organism after irradiation. First, eleven radiation emergencies characterized by chronic or protracted exposure of the human beings to ionizing irradiation were "revisited". The data provide evidence to suggest that at a daily exposure of about 10-100 mSv, humans are capable of coping with the excess cell loss for weeks or even many months without hematopoietic organ failure. Below 10 mSv/day, the organisms show some cellular or subcellular indicators of response. At dose rates above 100 mSv/day, a progressive shortening of the life span of the irradiated organism is observed. To elucidate the mechanisms relevant to tolerance or failure, the Megakaryocyte-thrombocyte cell renewal system was investigated. A biomathematical model of this system was developed to simulate the development of thrombocyte concentration as a function of time after onset of chronic radiation exposure. The hematological data were taken from experimental chronic irradiation studies with dogs at the Argonne National Laboratory, USA. The results of thrombocyte response patterns are compatible with the notion of an "excess cell loss" (compared to the steady-state) in all proliferative cell compartments, including the stem cell pool. The "excess cell loss" is a function of the daily irradiation dose rate. Once the stem cell pool is approaching an exhaustion level, a "turbulence region" is reached. Then it takes a very little additional stress for the system to fail. So an accurate, rapid and reliable techniques are important to asses the risks following exposures below 1 Gy.

## Conclusion

We think measuring RP by flow cytometry is a rapid, non-invasive method to asses an early affection of thrombopoiesis. This type of monitoring may be used as an indicator to detect early BM affection and to demand more controls in radiation protection.

## Competing interests

The authors declare that they have no competing interests.

## Authors' contributions

DS carried out the lab studies, participated in the sequence alignment, performed the statistical analysis and drafted the manuscript. MEA participated in the sequence alignment. SYA participated in the design of the study and. HG conceived of the study. All authors read and approved the final manuscript.
